# Serum Uric Acid and Lipid Levels in Patients With Acute Ischemic Stroke: A Cross-Sectional Study

**DOI:** 10.7759/cureus.28114

**Published:** 2022-08-17

**Authors:** SelvaKumar S Lokkanahalli, Nagappa H Handargal, Mishel M Papali, Nagarjun Subash

**Affiliations:** 1 Department of Internal Medicine, Ramaiah Medical College, Bangalore, IND

**Keywords:** stroke, risk factors, ischemia, hyperuricemia, dyslipidemias, cholesterol

## Abstract

Background and objective

Given the enormous public health burden posed by acute ischemic stroke (AIS), it becomes imperative to identify associated risk factors such as serum uric acid (SUA) and lipid profile that can be tangibly assessed. This could potentially provide useful markers for disease risk or progression, facilitating timely diagnosis and management of AIS. This study aimed to evaluate the SUA and lipid levels in patients with AIS.

Method

This cross-sectional study enrolled 66 AIS patients aged >18 years, from both genders. After a thorough medical history and clinical examination, each patient was subjected to SUA analysis and lipid profiling using an auto-analyzer with dedicated reagents. Results were statistically analyzed using Chi square test, and p-value ≤0.05 indicated statistical significance.

Results and interpretation

The study cohort showed a mean age of 61.17 ± 14.01 years and male to female (M:F) ratio of 1.7:1, with mean blood levels of SUA, triglyceride, and high-density lipoprotein (HDL) reaching 5.68 ± 1.71 g/dL, 205.42 ± 105.08 g/dL, and 29.80 ± 8.45 g/dL, respectively. Most patients suffered from hypertension (81.82%), diabetes (77.27%), and alcoholism (24.24%). Cerebrovascular Doppler findings revealed the combined presence of plaque and stenosis (24.24%). Male AIS patients showed a significantly greater association with alcohol and smoking/nicotine use (p<0.001). Gender showed no significant association with SUA, lipid profile, hypertension, and diabetes (p>0.05).

Conclusion

AIS is associated with hyperuricemia and dyslipidemia, with no significant gender differences.

## Introduction

Acute ischemic stroke (AIS), also called a cerebrovascular accident, is defined as an abrupt onset of a neurologic deficit that is attributable to a focal vascular cause that leads to cerebral ischemia. Its diagnosis depends on medical history, physical examination, and clinical and laboratory studies, including brain imaging [1]. It is one of the leading causes of adult disability and death worldwide, with greater prevalence in India as compared to that in high-income countries [2]. The cumulative incidence of stroke ranged from 105 to 152 per 100,000 persons per year, and the crude prevalence of stroke ranged from 44.29 to 559 per 100,000 persons in different parts of the country during the past decade [3]. The mortality rate of stroke in the acute phase is as high as 20%, and it remains high for several years after the acute event in comparison to the general population [4].

Given the high prevalence and significant morbidity/mortality associated with AIS, it becomes important to identify its risk factors and related disease indicators in order to provide timely management. Diabetes, hypertension, insulin resistance, smoking, obesity, hypercholesterolemia, and physical inactivity have been found to elevate the risk of suffering AIS. These risk factors are known to be associated with increased serum uric acid (SUA) level, which has also been reported as an independent predictor of stroke [4-6]. Hyperuricemia is also related to dyslipidemia, mainly owing to its association with the pathogenesis of atheroma, suggesting its role in AIS [7,8]. Hyperuricemia and impaired lipid profile are related to endothelial dysfunction and impaired vascular tone that could contribute to ischemic changes because they permit cerebrospinal fluid to cross the blood-brain barrier and cause areas of edema [9-12].

However, there is a paucity of scientific literature studying both, hyperuricemia and impaired lipid profile, in AIS patients. Identifying the changes in these profiles could aid in the timely diagnosis and management of such patients and help formulate targeted efforts to mitigate the risk of AIS. Therefore, the present study was conducted to evaluate the SUA and lipid profile in patients with AIS.

## Materials and methods

This cross-sectional study was conducted at a tertiary care hospital in Bengaluru, India, from October 2018 to September 2020, after obtaining ethical clearance from the institutional review board. The present study included patients aged >18 years, irrespective of their gender, diagnosed with AIS based on clinical and radiological evaluation, including computed tomography (CT) and magnetic resonance imaging (MRI) of the brain, after obtaining written informed consent from them. The study also excluded patients with the chronic intake of drugs causing hyperuricemia, patients on treatment for hyperuricemia, and patients with conditions that alter serum uric acid levels (lymphoproliferative diseases, polycythemia, myeloproliferative disorders, chronic renal insufficiency, malignancy, diabetic ketoacidosis, and lactic acidosis).

The sample size was calculated based on a previous study conducted by Arora et al. [13]. Considering a confidence level of 95% and power of 80%, to detect a minimum difference of 0.8 g/dL with an absolute precision of 0.8%, the minimum required sample size was found to be 56 patients, as per the formulae:

n = [2S^2^p {z(1-α/2) + z(1-β)}]^2^/[µ^2^d],

wherein S^2^p = (S1^2^ + S2^2^)/2,

where n = minimal sample size, S1^2^ = standard deviation in the first group, S2^2 ^= standard deviation in the second group, µ^2^d = mean difference between the samples, α = significance level, 1-β = power.

Accordingly, 66 patients were included in this study. After thorough history taking, a routine clinical examination of all systems, as well as measurements of blood pressure, pulse rate, respiratory rate, body temperature, and oxygen saturation, was performed. Following this, a fasting blood sample (3-5 mL) was taken from each patient using a sterile syringe and needle and subjected to uric acid analysis and lipid profiling employing Cobas 6000 auto-analyzer (Model no: c501, Roche diagnostics, Germany) with dedicated reagents. Serum uric acid (SUA) levels were assessed by enzymatic (uricase) calorimetric method (Roche Diagnostics, GmbH, Mannheim, Germany), triglyceride (TG) levels by enzymatic (lipase) calorimetric method, total cholesterol (TC) by enzymatic (cholesterol esterase) calorimetric method, high-density lipoprotein (HDL) by direct measure, low-density lipoprotein (LDL) by direct measure, and very low-density lipoprotein (VLDL) by triglyceride percentage.

For SUA, the lower limits of detection were 0.2 mg/dL (range: 0.2-25.0 mg/dL), and intra-assay and inter-assay coefficients of variation were equal to 0.5% and 1.7%, respectively. Hyperuricemia was defined as a serum urate concentration >7.5 mg/dL (450 µmol/L) in men and >6.2 mg/dL (372 µmol/L) in women, in agreement with the normal value provided by the clinical laboratory.

Statistical analysis

Data were compiled and analyzed using statistical software R version 4.0.3 and Microsoft Excel (Microsoft Company, USA). Continuous variables are represented in mean ± standard deviation (SD) form and categorical variables by a frequency table. QQ plot/Shapiro-Wilk’s test was used to check the normality of variables. Independent sample t test/Mann-Whitney U test was done to compare two groups. Chi square test has been used to assess the association between two categorical variables. A p-value ≤ 0.05 indicates statistical significance.

## Results

The study consisted of 66 patients out of which 40 (60.6%) were males and 26 (39.4%) were females with a mean age of 61.17 ± 14.01 years and a male to female (M:F) ratio of 1.7:1. Tables 1, 2 present the descriptive statistics for continuous and categorical variables. The mean serum uric acid levels in the studied patients were 5.68 ± 1.71 mg/dL, and 21 patients (31.81%) had hyperuricemia. The mean serum TG and HDL were found to be 205.42 ± 105.08 g/dL and 29.80 ± 8.45 g/dL, respectively. High LDL, cholesterol, and triglyceride were observed in 46 (69.69%), 48 (72.72%), and 44 (66.66%) patients, respectively, and 42 patients (63.63%) had low HDL. The study showed that 21 patients had both hyperuricemia and dyslipidemia with no significant gender disparity. Most patients suffered from diabetes (77.27%), hypertension (81.82%), and alcoholism (24.24%). Cerebrovascular Doppler findings revealed the combined presence of plaque and stenosis (24.24%).

**Table 1:** Descriptive statistics for continuous variables IQR: interquartile range; SD: standard deviation.

Variables	Mean (SD)	Median (IQR)
Age (years)	61.17 (14.01)	61.50 (54.25-70.75)
Serum uric acid (mg/dL)	5.68 (1.71)	5.55 (4.40-6.60)
Total cholesterol (mg/dL)	218.61 (45.90)	214 (198.50-243.75)
Triglycerides (mg/dL)	205.42 (105.08)	195.50 (148.50-242.25)
High-density lipoprotein (mg/dL)	29.80 (8.45)	27 (24-33.75)
Low-density lipoprotein (mg/dL)	151.45 (33.89)	148 (131.25-176.75)
Very low-density lipoprotein (mg/dL)	44.20 (14.15)	43.50 (38-48)
Homocysteine (µmol/L)	16.75 (9.64)	14.12 (8.89-21.98)

**Table 2:** Descriptive statistics for categorical variables

Variables	Subcategory	Frequency	Percentage (%) (n=34)
Gender	Female	26	39.39%
	Male	40	60.61%
Age groups (years)	21-35	2	3.03%
	36-50	13	19.70%
	51-65	24	36.36%
	66-80	22	33.33%
	>80	5	7.58%
Diabetes	No	15	22.73%
	Yes	51	77.27%
Alcohol	No	50	75.76%
	Yes	16	24.24%
Hypertension	No	12	18.18%
	Yes	54	81.82%
Cerebrovascular Doppler finding	Normal	35	53.03%
	Plaque	11	16.67%
	Plaque and stenosis	16	24.24%
	Stenosis	3	4.55%
	Vertebral artery ostium	1	1.52%

Table 3 presents the distribution of subjects based on gender and risk factors. As per Chi square test, gender shows a statistically significant association with alcohol and smoking/nicotine use (p<0.001), but not with diabetes and hypertension (p>0.05).

**Table 3:** Distribution of subjects based on gender and risk factors

Variables		Gender				p-value
		Female (n=26)		Male (n=40)		
Alcohol	No	26	100%	24	60%	<0.001*
	Yes	0	0%	16	40%	
Smoking/nicotine	No	25	96.15%	13	32.5%	<0.001*
	Yes	1	3.85%	27	67.5%	
Diabetes	No	9	34.62%	6	15%	0.119
	Yes	17	65.38%	34	85%	
Hypertension	No	5	19.23%	7	17.5%	0.999
	Yes	21	80.77%	33	82.5%	

Table 4 presents a gender-wise comparison of various parameters. Using Mann-Whitney U test, gender showed no significant association with SUA, TC, TG, HDL, LDL, and VLDL levels (p>0.05).

**Table 4:** Gender-wise comparison of various parameters IQR: interquartile range; SD: standard deviation.

Variables	Female (n=26)		Male (n=40)		p-value
	Mean (SD)	Median (IQR)	Mean (SD)	Median (IQR)	
Age (years)	64.62 (15.76)	68.5 (55.75-73.75)	58.93 (12.45)	60 (53.25-66.70)	0.055
Serum uric acid (mg/dL)	5.18 (1.24)	5.1 (4.43-5.9)	6 (1.89)	6 (4.4-7.18)	0.093
Total cholesterol (mg/dL)	212.35 (45.25)	209.5 (201.25-232.25)	222.68 (46.42)	215.5 (196.25-260.50)	0. 355
Triglycerides (mg/dL)	195.15 (111.65)	180.5 (134.25-205.25)	212.1 (101.48)	198 (164.25-256)	0.234
High-density lipoprotein (mg/dL)	30.85 (9.95)	27 (24-34.75)	29.13 (7.37)	27 (23.75-33.25)	0. 655
Low-density lipoprotein (mg/dL)	153.38 (27.12)	149 (137-168)	150.2 (37.94)	147.5 (130.75-181.5)	0.859
Very low-density lipoprotein (mg/dL)	41.65 (10.48)	44 (37.25-45)	45.85 (16.07)	43 (39-51.25)	0.396

## Discussion

Given the enormous personal and public health burden posed by AIS, it becomes imperative to identify associated risk factors that can be tangibly assessed. SUA and lipid levels are a few of such measurable factors that have been found to be associated with AIS and could provide useful markers for disease risk or progression. With this vision, the present study was conducted to evaluate the SUA and lipid levels in patients with AIS. This study showed that dyslipidemia was seen in all 66 (100%) patients and all of them had more than one lipid parameter deranged. Mean serum uric acid levels in the studied patient were 5.67 ± 1.68, and 21 patients (31.81%) had hyperuricemia.

This study resonates with the findings of Arora et al., who studied a similar population with a mean age of 63.2 ± 14.8 years and found the mean SUA levels to be 5.5 ± 01.7 g/dL [13]. Another research team comprising Mehrpour et al. studied 55 AIS patients with an M:F ratio of 1.2:1 and a mean age of 67 ± 14 years and found the mean SUA levels to be 5.94 ± 1.70 mg/dL with hyperuricemia afflicting 47.3% of the patients [14]. The prevalence of raised SUA levels in AIS patients could be explained by its association with endothelial dysfunction, impaired vascular tone, and atheroma formation [9-12]. Even a mild elevation of SUA can be associated with cerebral ischemia in adults as it permits cerebrospinal fluid to cross the blood-brain barrier and cause areas of edema [9-12]. Mehrpour et al. also found SUA levels to be significantly higher in males than in females (p=0.04), while this difference was not significant in the current study (p=0.09) [14]. This lower level in women apparently reflects estrogen-related enhancement of renal urate clearance [15].

Dyslipidemia observed in the present study was also mirrored in the work by Yuan et al. who noted a significant rise in TG, TC, and low-density lipid cholesterol (LDL-C) with a significant fall in high-density lipid cholesterol (HDL-C) levels in AIS patients and suggested that an adverse lipid profile, especially elevated LDL and TC, is an independent risk factor for large artery atherosclerotic cerebrovascular accident [16]. Moreover, Son et al. demonstrated that SUA levels showed a positive association with serum TC, TG, and LDL-C levels and an inverse relation with serum HDL-C levels [17]. This relationship between SUA and adverse lipid profile could be owed to hyperuricemia promoting LDL-C oxygenation, lipid peroxidation, vascular smooth cell proliferation, platelet adhesiveness, and synthesis of proinflammatory factors like monocyte chemoattractant protein-1, interleukin-lβ, interleukin-6, and tumor necrosis factor-alpha (TNF-α), thus promoting atherosclerotic arterial occlusion [9-12]. This suggests that hyperuricemia and dyslipidemia could possibly serve as measurable characteristics of AIS. Similarly, Ali et al. concluded that the mean SUA level was significantly higher in males (317 ± 90 μmol/L) than in females (255 ± 65 μmol/L) (p<0.001) and showed a significant linear association with an adverse lipid profile (p<0.01 for trend) [18].

The presence of other risk factors for stroke like smoking, alcohol, hyperhomocysteinemia, diabetes, and hypertension, as seen in the present study, has also been confirmed by other researchers [19-21]. In our study, 16 (24.24%) male patients had a history of alcohol consumption (p<0.001) and 28 (42.42%) patients were smokers/nicotine consumers out of which one (1.5%) was female and 27 (40.9%) were males (p<0.001). There was a statistically significant difference found between female and male with respect to alcohol and smoking/nicotine consumption. With respect to other risk factors, diabetes mellitus was found in 37 (56.06%) out of which 24 (36.36%) were males and 13 (19.69%) were females. Fernandes et al. evaluated carotid arteries in stroke patients using color Doppler sonography and found the highest incidence of stroke among males aged 60-69 years with concomitant risk factors like hypertension, diabetes mellitus, smoking, and family history [22]. Of 50 patients, over 60% showed significant stenosis and 78% showed atherosclerotic plaque features that were also noted in the present research. Hence, we can confidently say that according to this study, AIS is associated with hyperuricemia and dyslipidemia, with no significant gender differences. Hyperuricemia can also be used as an early predictor for AIS and be used as a monitoring tool for stroke prevention.

## Conclusions

Conclusively, the present study establishes the association of hyperuricemia and dyslipidemia with AIS. This can potentially be utilized to assess at-risk individuals and intervene early at various systemic levels to minimize AIS-related morbidity and mortality. However, the present research was conducted at a single medical center with a small sample size and as a cross-sectional design with no control group. Further multicentric longitudinal studies are encouraged with a larger sample size to confirm these parameters as markers of AIS. This could revolutionize policy-making, resource allocation, and therapeutic options in managing patients at risk of AIS.
